# Factors associated with the high prevalence of myopia and its decrease—A historical review

**DOI:** 10.1111/aos.70001

**Published:** 2025-10-06

**Authors:** Olavi Pärssinen

**Affiliations:** ^1^ Gerontology Research Centre and Faculty of Sport and Health Sciences University of Jyväskylä Jyväskylä Finland

**Keywords:** epidemic of myopia, font size, near work, outdoors, reading distance

## Abstract

**Purpose:**

To review historical and recent studies on the epidemiology of myopia, identify the factors associated with its prevalence and determine how myopia has been successfully prevented.

**Methods:**

Historical and recent publications were reviewed regarding factors associated with the prevalence of myopia and the success of myopia prevention. Among the historical studies, Herman Cohn's studies in Germany and Johan Widmark's studies in Sweden were referenced and compared.

**Results:**

In the 1800s, the prevalence of myopia caused by school glasses was high in the upper grades of many schools in Germany and Sweden. In Germany, the mean prevalence of myopia in the upper grades of 24 schools was 58%, exceeding 70% in several schools. At the same time in Sweden, the corresponding average prevalence was 45%, with the highest prevalence of 65%. Germany and Sweden implemented several generally accepted reforms to reduce the educational burden of schooling. Widmark compared the prevalence of myopia in the same schools in Sweden between the periods 1822–1883 and 1904–1905. The mean myopia prevalence decreased from 22.2% to 9.8%, and from 45.3% to 17.4% in the highest grades of these schools. Widmark identified three factors to explain the decrease in myopia prevalence: (1) improved lighting due to electric lights; (2) Fraktur fonts in schools were replaced by Antiqua; and (3) the number of outdoor activities was increased. The discussion aims to find similarities between Cohn's and Widmark's studies and the current myopia boom and to describe how these historical observations might currently be used to prevent myopia.

**Conclusion:**

Myopia generally begins after starting school. The younger the children are subjected to the strain caused by near work and the less time spent outdoors at school, the higher the prevalence of myopia and the higher the myopia in adulthood. To control the current myopia boom in countries where the prevalence is highest, major changes should be implemented in the education system to reduce the need for near work and increase the time children spend outdoors.

## 1 INTRODUCTION

Myopia, that is near‐sightedness, has been known since ancient times from the texts by Aristotle (384–322 BC). The terminology myopia is derived from the Ancient Greek: μυωπία, muōpía, ‘shortsightedness’, from μύω (múō, ‘to shut, squint’) + ὤψ (ṓps, ‘eye’)+ ‐ία (Curtin, 1985). Aristotle identified this near work as the aetiology of myopia and described the symptoms of uncorrected myopia. Kepler (1611) was perhaps one of the first to suggest that excessive near work is a determining factor in the aetiology of myopia. He went on to propound a ‘near work’ hypothesis for myopia by stating that studying and fine work in childhood rapidly accustoms the eye to near objects. With advancing years, this adaptive mechanism results in a permanent, finite far point such that distant objects can only be seen poorly. Thereafter, numerous studies in the 19th century established connections among education, near work and myopia (Cohn, 1882; Tscherning, 1883; Widmark, 1909).

In the 1900s, the opinion came to the fore that the cause of myopia is mainly hereditary. Sorsby (1933) stated that ‘the optimism of those who regarded myopia as a product of controllable environmental conditions have not proved altogether justified’. This view was supported, for example by Sorsby et al. (1966) and Keller (1973), who highlighted questions, especially based on studies in Inuit populations (Morgan et al., 1975). The prevalence of myopia in Inuits increased drastically from a low level of less than 3% to a level of more than 50% in generations of young adults after the Second World War. This could not be explained without considering the influence of environmental factors. The author's doctoral dissertation (Pärssinen, 1986) showed that more near work time and less outdoors were associated with myopia in 11‐ to 12‐year‐old children. A later analysis of the data of 7‐, 11‐, and 15‐year‐olds from the same study showed that myopic parents, greater near work time, less outdoors time, and a higher near work/outdoors ratio were the main factors increasing the odds for myopia (Pärssinen & Kauppinen, 2022). Myopia was rare in 7‐ and 11‐year‐olds if daily near work at home did not exceed one hour or if the near work/outdoors ratio was not higher than 0.5. In the study of Jones et al. (2007), lower amounts of sports and outdoor activity increased the odds of becoming myopic in those children with two myopic parents compared to children with either zero or one myopic parent, whereas the chance of becoming myopic was lowest for children with no myopic parents with the highest amount of sports and outdoor activities. In their study, significant protective associations with increased outdoor activities were seen as the lowest (*p* = 0.04) and middle (*p* = 0.02) tertiles of near‐work activity. Morgan and Rose (2005) reported that while there may be small genetic contribution to school myopia, environmental factors, increased education, less time spent outdoors, and urbanization are the major factors increasing the prevalence of myopia around the world. The genetic studies have identified several loci associated with refractive error and myopia (Tedja et al., 2018; Tideman et al., 2021). However, genetic factors have only been able to partially explain the prevalence of myopia (Tedja et al., 2018). Thus, it is believed that a shared living environment partly explains the connection between myopia in children and their parents (Fan et al., 2016). Studies carried out in the late 1900sand the present millennium have undeniably shown that the most important factors associated with myopia are considerable near work time, little time spent outdoors, which factors are related to the general increase of education (Pärssinen, 1986, 1987; Pärssinen & Kauppinen, 2022; Rose et al., 2008; Sperduto et al., 1983). The increase in myopia in recent decades, especially in East and Southeast Asian countries, can be characterized as an epidemic such that in East Asian countries, 80%–90% of school‐leavers are affected by myopia, and 10%–20% of them have high myopia, which is connected with an increased risk for sight‐threatening pathologies (Morgan et al., 2012). The WHO report (2016) estimated that myopia will affect 1.893 billion people (i.e. 27% of the world's population) by 2020. The characteristics described in myopia studies of the 1800s and the beginning of the 20th century have many similarities with today's myopia boom, which is especially present in East and South Asian countries. In the 1800s, a high prevalence of myopia was reported in German (Cohn, 1882) and Swedish (Widmark, 1909) schools. Several measures were implemented to reduce the incidence of myopia. As a result, the high prevalence of myopia among Swedish schoolchildren decreased significantly over approximately 20 years. This article aims to review these historical studies and clarify how the findings and measures of past times can be applied to prevent the current increase in myopia prevalence.

## 2 MATERIALS AND METHODS

This article reviews Cohn's studies (1882) on the high prevalence of myopia in German schools in the late 1800s and his views on the factors that caused the high prevalence of myopia, especially in the upper grades of schools. Widmark's study (1909) from Sweden reported a decrease in myopia In Swedish schools in the early 1900s and presented explanations which is why the prevalence of myopia in schools decreased. The results of these two studies aim to find explanations why there are large differences in the prevalence of myopia today.

## 3 REVIEW OF HERMAN COHN'S AND JOHAN WIDMARK'S STUDIES

### 3.1 Herman Cohn

The German ophthalmologist Hermann Cohn (Figure 1) was born in 1838 in Breslau and died in 1906 also in Breslau (Cohn, n.d., Wikipedia 1). He attended the Maria Magdalene Gymnasium in Breslau. There, in the mid‐1800s, he learned how inadequate classroom lighting was. Throughout winter, afternoon lessons probably had to be held under artificial lighting, often also during early morning hours. A candle was placed on the school desk between two seats during writing lessons but not during reading lessons.

**IGURE 1:**
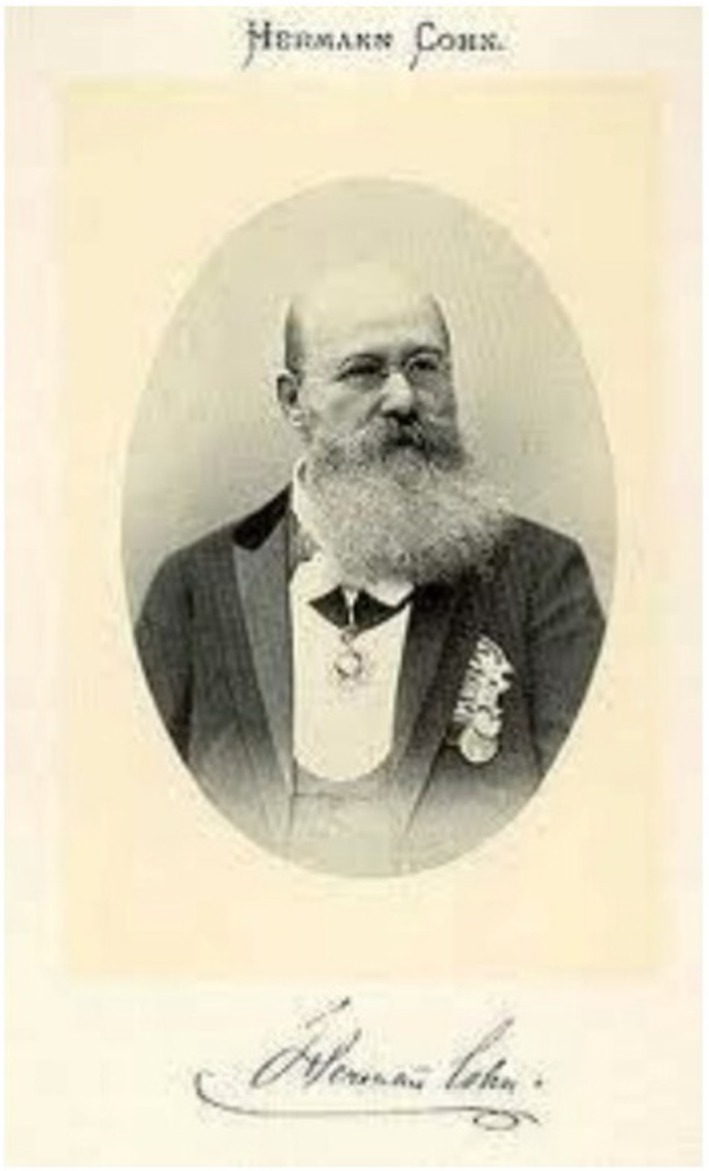
Picture of Herman Cohn (https://de.wikipedia.org/wiki/Hermann_Cohn_(Mediziner)).

Cohn received his medical doctorate in 1863 in Berlin, worked in 1864 as Richard Förster's assistant physician in Breslau, and continued in 1866 his studies with the famous ophthalmologists Albrecht von Graefe in Berlin, de Wecker in Paris and Ritter von Arlt in Vienna. He established his own eye clinic in Breslau and promoted a lot, especially for young people's eye heath care. Cohn was appointed as an assistant professor in Breslau. His books, special lectures, and publications made him one of Germany's leading ophthalmologists and earned him a lot of praise. In 1888, he was elected a member of the Leopoldina Academy of Sciences.

Hermann Cohn maintained his ophthalmological clinic with his own funds, and the poor were treated free of charge. He treated 45,000 patients, performed approximately 5,000 major surgeries and restored the sight in tens of thousands of people (Cohn 1882). Cohn invented and improved ophthalmologist tools, such as goggles, light testers, school lamps, eye tests and colour charts. In the myopia section of his book, “Herman Cohn. Lehrbuch Der Hygiene Des Auges” (Cohn, 1882), he referred to his and other researchers' studies on the prevalence of myopia in Germany and other countries since the mid‐1800s, extensively discussing the aetiology, pathogenesis, prevention and treatment of myopia.

### 3.2 Review of Herman Cohn's textbook ‘Lehrbuch Der Hygiene Des Auges’ (Cohn 1882)

**FIGURE 2:**
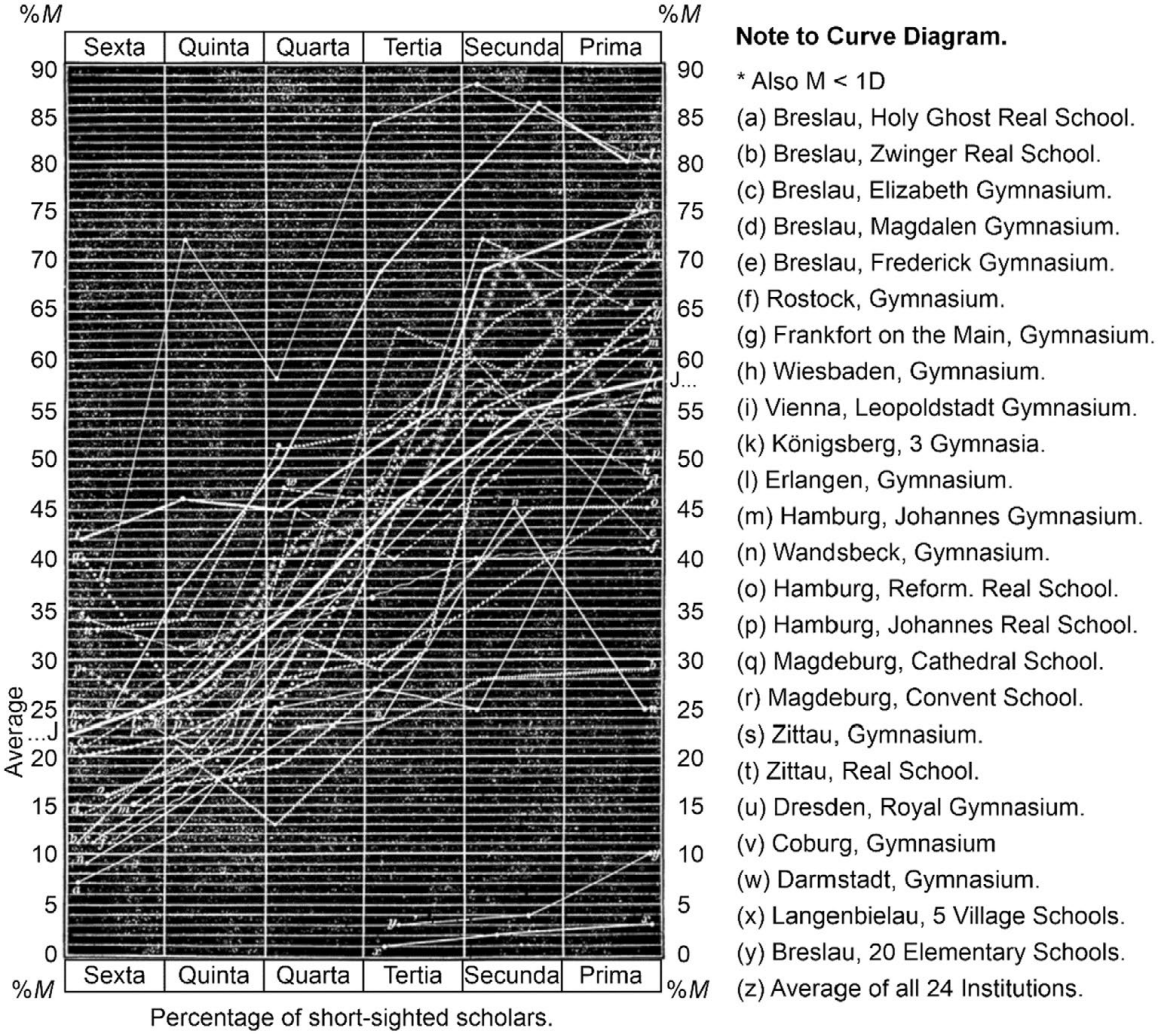
Curve diagrams showing the increase of myopia from class to class in 24 German gymnasiums and real schools. Copy from Cohn page 72.

Beginning in 1865/1866, Cohn examined 10,060 schoolchildren. In that study, he found a myopia prevalence of 44.0% in the highest grade of grammar schools and 55.8% in the upper grades of secondary schools, although Cohn did not include in his statistics either myopia degrees lower than −1 D or cases in which myopia occurred in only one eye. Typically, the degree of myopia increased when pupils moved to higher grades; however, myopia substantially varied among schools. The three‐page supplement shows the prevalence rates of myopia in different schools in Germany during the 1800s (Table S1). The mean prevalence rates of myopia in the three highest grades of more than 100 schools were 45%, 55% and 58%. As an example of a high myopia prevalence, Cohn referred to Gartner's studies on theological students from Tübingen. Between 1861 and 1882, the prevalence of myopia at different theological faculties varied between 78% and 81%. By contrast, in village and municipal schools, the prevalence of myopia was very low. For example, in five village schools in Breslau (1,486 pupils), the total prevalence rates of myopia were 1.4%, 2.0% and 3.0% in the three highest grades, and in 20 municipal schools (4,978 pupils), the prevalence of myopia was 6.7%, with 1%, 4%, and 10% in the three highest grades (Table S1). Figure 2 shows the prevalence of myopia in different schools by grade. The average age in the highest grade, ‘Prima’ corresponds to approximately 18 years.

Cohn developed six theories to explain the development of myopia: (1) heredity, (2) accommodation, (3) convergence, (4) nerve drag, (5) extraocular muscles and (6) near work (Cohn 1892). He largely refuted the hereditary theory because myopia was only 10% more frequent in his patients when both parents were myopic. Cohn stated that most ophthalmologists believe that continuous near work, with the head tilted forward and with poor illumination, stimulates myopia. Cohn considered poor lighting to be the leading contributing factor to the overworking of the eyes in schoolchildren and the incidence of myopia. At school and home, children had to study by candlelight. Moreover, textbook printing was poor, mostly using the Antiqua font, and the printed text was difficult to read. This kind of text was frequently read at school, especially in the language classes. Reading this type of text was slow and required a shorter reading distance, especially under bad lighting conditions.

**FIGURE 3:**
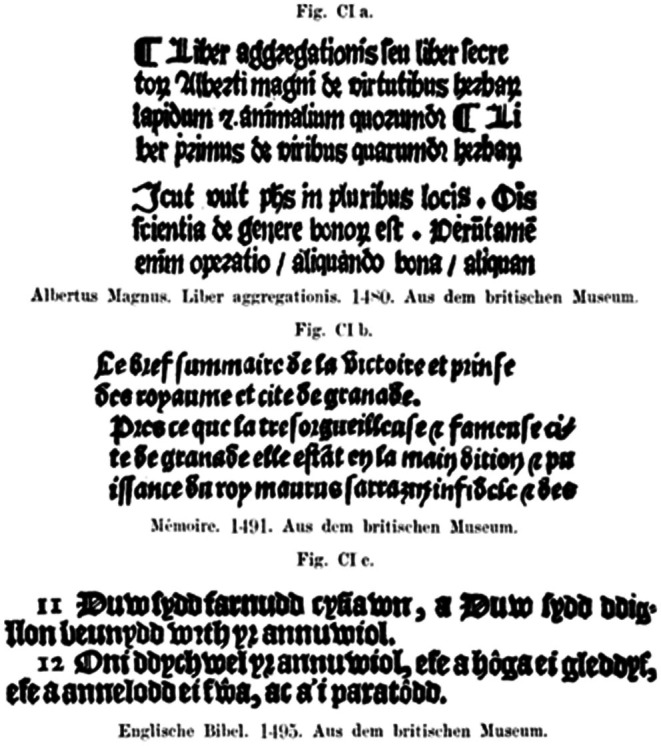
A sample of texts needed to read. The copy from p. 454 from Cohn (1892) (https://books.google.fi/books?id=Vob‐GKJfkUcC&printsec=frontcover&hl=fi#v=onepage&q&f=false).

Figure 3 illustrates examples of such texts. In many textbooks, especially those for classical languages and French, the font was 1.25 or 1 mm high, and in atlases, the font height was as low as 0.5 mm. Cohn also drew attention to how narrower spaces between letters and words, as well as smaller font sizes, make the text more difficult to read. Cohn recommended a text size of 2.5 mm for schoolbooks.

The more detailed the writing was, the closer the children had to look. The two pictures in Cohn's book characterize children's head positions and reading distances when writing two different types of font: tilted font (Schiefschrift) or non‐tilted, text‐type font (Steilschrift).

**FIGURE 4:**
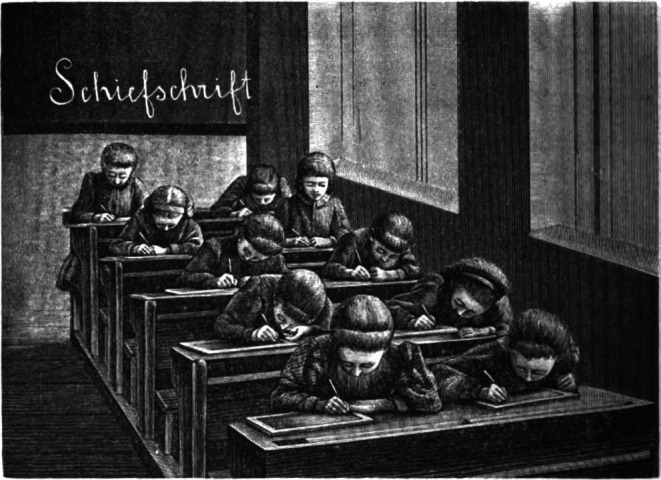
The girls' writing penmanship (Schiefschrift) needs more accurate focusing and shorter viewing distance (Cohn p. 428).

**FIGURE 5:**
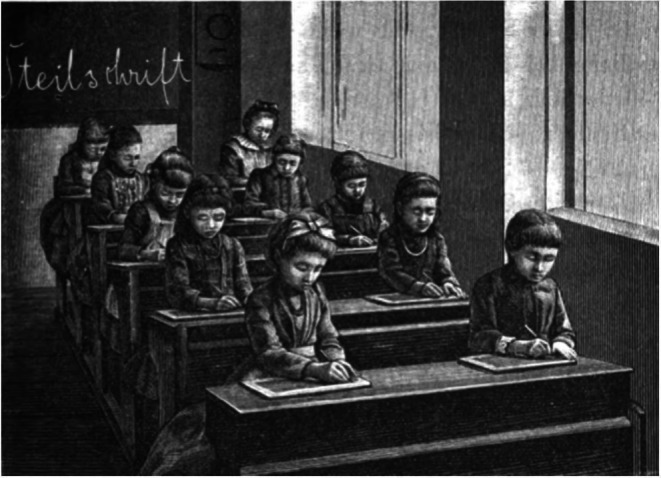
The girls’ writing non‐tilted, text‐type font (Steilschrift)) don't need accurate focusing and short viewing distance (Cohn p. 428).

Cohn concluded that writing in tilted text fonts is a cause of a shorter reading distance, which is a risk factor for myopia. Figures 4 and 5 characterize the postures of schoolchildren and the distances of near work in the two writing tasks, which require different levels of precision.

**FIGURE 6:**
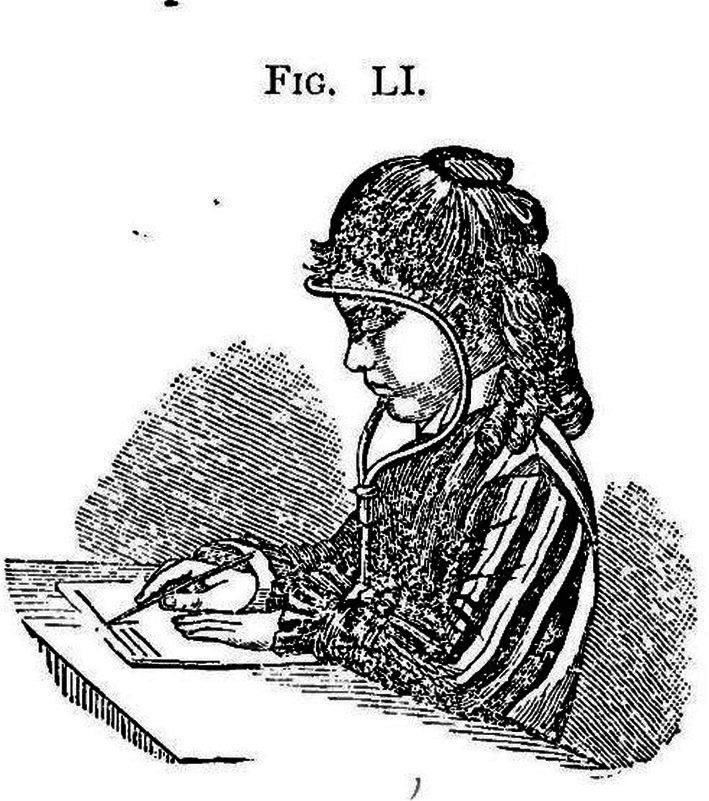
One example of different devices that were used to keep a sufficient distance when reading or writing (Cohn p. 344).

A short distance of viewing was commonly regarded as one of the main risk factors for myopia. Therefore, various aids have been developed to prevent an overly close viewing distance. Figure 6 shows an example of the many devices used to maintain an appropriate reading distance.

As a general recommendation for schoolchildren, Cohn proposed that writing exercises should never be extended to more than 2 h and writing should only occur at the brightest time of the day, at noon. He also recommended that after near work for an hour, a 15‐min break during which the children had to go outside. He believed that after 5–6 h of school, it would be most detrimental if pupils still had to do homework. Sundays and holidays should be resting times for the eyes. If myopia progresses, one should not touch a book, at least during the summer vacation.

Myopia and related complications have been a common concern for ophthalmologists since the mid‐1800s. Therefore, in 1881, a consensus meeting was held in Strasbourg. It was the lasting credit of Field‐Marshal von Manteuffel, the Lord‐Lieutenant of Alsace‐Lorraine, that he, unsolicited, convoked the commission of eminent medical professors to draw up a report on school hygiene. This valuable report was published in September 1882. The first paragraph of the Strasbourg Manifesto 1882 about how to arrange school education focused on its content. Table 1 summarizes the first paragraph of this manifest, recommendations of the Strasbourg Commission for the organization of weekly school education (The whole manifest in Supplement A).

**TABLE 1:** Paragraph 1 of the Strasbourg Manifesto about the arrangement of school education, focusing on its weekly content.

Age years	Grades of school	Teaching hours	Singing hours	Gymnastic hours	Homework hours	Total
7 and 8	1 and 2	18	1	2–2.5	3	24.0–24.5
9	3	20	1	2–2.5	5–6	28–29.5
10 and 11	4 and 5	24	2	2–3	8	36–37
12, 13, 14	6 and 7	26	2	2	12	42
15 to 18	8 and 9	30	2	2	12–18	46.0–52.0

*Note*: Quoted and edited from Cohn (1882), p. 504.

From the sane manifesto:

Paragraph 2. Between every two lessons, whether in the morning or afternoon, there should be a break of 10 min. If more than two lessons are consecutive, there should be a break of 15 min between the 2nd and 3rd lesson and a break of 20 min between the 4th and 5th lesson.

Paragraph 10. Swimming practice, outdoor games, excursions, and skating are strongly recommended, in addition to the obligatory gymnastics lessons. Altogether, 8 h per week should be devoted to physical exercise. The entire 24‐point manifest is provided in Supplement A.

### 3.3 Myopia in Sweden at the turn of the 1800s and 1900s

At the end of the 1800s, myopia was also common in the upper grades of Swedish schools. When comparing the myopia prevalence rates approximately 20 years later, Professor Widmark (1909) found a significant decrease in myopia in the same schools. A comparison of the results of the above studies by Cohn in Germany with those of Widmark in Sweden can help understand the causes of myopia and identify the methods that can be used to reduce myopia.

### 3.4 Erik Johan Widmark

**FIGURE 7:**
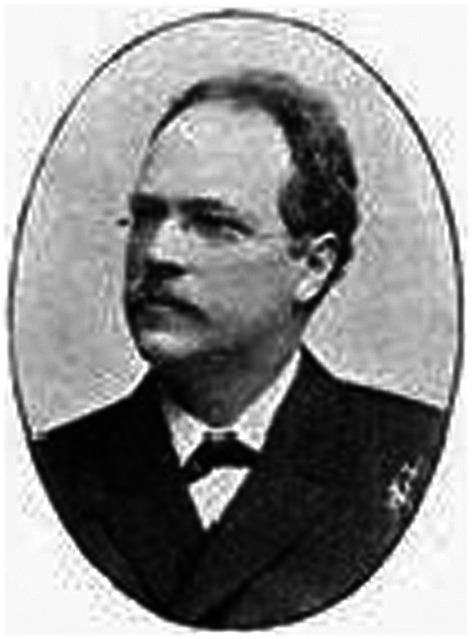
Johan Widmark. (Widmark, n.d., Wikipedia, photo)

Erik Johan Widmark (1850–1909) (Figure 7) was the first appointed professor of ophthalmology in Sweden at the Karolinska Institute, Stockholm, from its establishment in 1889 to his death in 1909 (Widmark, n.d., Wikipedia).

Widmark's research topics included the physiology and bacteriology of the eye and the influence of UV radiation on the lens and retina. In addition, he conducted influential studies on myopia. His publication (Widmark, 1909) describes the high prevalence of myopia among schoolchildren in the Stockholm area in the late 1800s and the decline in myopia prevalence over approximately 20 years during the transition to the 1900s.

### 3.5 Review of Johan Widmar's study ‘Über die abnahme der kurzsihtigkeit in den höheren knabenschulen Swedens’ (Widmark, 1909)

Widmark compared the myopia prevalence in several schools in the Stockholm area and in different areas in Sweden between the periods 1882–1884 and 1904–1905. The prevalence of myopia in Swedish schools was quite high, in the two highest grades in the period 1882–1884, although it did not reach the same level as that in many German schools. Over approximately 20 years, the prevalence of myopia was more than halved. Table 2 compares the changes in myopia prevalence among several Swedish schools. The average prevalence of myopia in these schools decreased by more than half during 20 years, from 22.2% to 10.8%.

**TABLE 2:** Myopia prevalence in some Swedish schools from Stockholm and surrounding areas.

Year	Prevalence of myopia in the entire school				Prevalence of myopia in the two highest grades of the school	
	1882–1884		1904–1905		1882–1884	1904–1905
Region	*N*	%	*N*	%	%	%
Stockholm and Norra						
Latin grammar school	569	23.9	713	15.4	52.0	31.0
Real school	300	31.0	622	7.7	43.2	12.3
New elementary school	286	24.0	375	10.7	50.0	11.8
Higher general schools						
Uppsala	585	20.8	546	14.4	40.1	15.0
Linköping	488	26.0	588	11.4	50.5	28.3
Skara	389	10.8	434	5.5	25.3	7.1
Falun	223	19.7	352	6.2	65.4	14.7
Luleå	91	20.0	248	6.9	46.6	12.1
Sundsvall	178	17.0	363	7.7	30.0	27.2
Higher schools in Stockholm and Uppsala	3973	28.3	3587	12.6	52.0	19.3
10 schools in different parts of Sweden	3054	23.2	4501	9.8	43.2	12.2
Mean prevalence of myopia in all of the above schools, %, mean (SD), range	22.2 (5.53), 10.8 to 30.1		9.8 (3.35), 5.5 to 15.4		45.3 (11.01), 25.0 to 65.0	17.4 (11.01), 7.0 to 31.0

*Note*: Translated and edited from Widmark (1909): ‘The figures for 1905 were partly taken from the Official Statistics of Sweden, 1905 and partly compiled from primary data from the annual catalogues of individual schools. I obtained some of these paragraphs thanks to the kindness of the Chancellery Secretary, Mr. Gustafsson’ (Widmark, 1909).

In the highest two grades during the period 1882–1884, the prevalence of myopia was 50% or higher in several schools. The average prevalence of myopia across all schools decreased over 20 years, from 22.2% to 9.2%. Similarly, the average prevalence of myopia in the two highest grades decreased from 45.3% to 17.4%. When examining the changes on a school‐by‐school basis, the prevalence of 65.4% in the two highest grades decreased to 14.7% in the New Elementary School in Stockholm, that in Luleo from 50.0% to 11.8%, and that in Falun from 65.4% to 14.7%.

### 3.6 Factors related to decrease in myopia prevalence

Widmark presented three factors which, in his opinion, were responsible for the significant decrease in the prevalence of myopia over the past 20 years.

**The first** and most important reason he considered was the significant improvement in the lighting and quality of printing, making near work and reading easier both at school and at home. Electric lights had become common, the Fraktur font had been replaced almost entirely by the Antiqua font, and the quality of textbook printing had significantly improved.

**The second** of Widmark's explanations for the decrease in myopia prevalence was a decrease in the study of classical languages, for example in Latin schools. In general, one obviously had to read more during language lessons, and the constant search for vocabulary in dictionaries with their fine print strained the eyes. An example of this is the Greek language with its completely different alphabet. As language learning became less common, learning focused more on subjects such as mathematics, physics and chemistry. Widmark suggested that the difference in the prevalence of myopia among schools depends on the time required for reading.

**As a third point**, Widmark emphasized that the reason for the decrease in myopia was the increase in sports and physical exercises, especially outdoor games, both at school and during spare time. To support this observation, Widmark referred to the prevalence in two higher secondary schools, the Djurholm and Lundberg schools, during the period 1903–1908. In those schools, special attention was paid to increasing exercise and sports hours at school and during leisure time. The myopia prevalence in the two highest grades in these schools was 9.3%, whereas the prevalence in the same grades in other schools of Stockholm was 15.9–18.7%.

## 4 DISCUSSION

In their reports, Cohn and Widmark described a high prevalence of myopia in Germany and Sweden in the 19th century in the highest grades of many schools. In many schools, myopia was nearly as prevalent as it is now in school leavers in many Asian countries (Morgan et al., 2012). In Germany and Sweden in the 1800s, there was no compulsory education and only a small part of the population completed the highest level of basic education. There is very little epidemiological data about the population‐based prevalence of myopia at that time. The epidemiological study of Tscherning (1883) from Copenhagen characterizes the connection of education with this matter. In his study myopia was common among students, 32%, whereas rare in peasants, 2% (Tscherning, 1883). Presumably, the prevalence at the population level at that time was a few percent. Since the Second World War, the level of education has risen globally, and this is one of the basic reasons for the increase in myopia. For example, in Finland, the prevalence of myopia in the adult population born before the Word War II was less than 10% and since then the prevalence has stabilized at about 25% (Pärssinen, 2012). In several Asian countries, the prevalence of myopia among school leavers is now about 80% or more (Morgan et al., 2012). The probable reasons for the large differences in the prevalence of myopia in different countries can be found in differences in the intensity of education, differences in near work and outdoor activities. Awareness of these things in the late 1800s led to the 24‐point Strasbourg Manifesto on the arrangement of school education established in 1882 in cooperation with Cohn and other German authorities.

Sweden implemented school reforms in accordance with the recommendations of the Strasbourg Manifesto. Widmark showed that within 20 years, myopia prevalence in the same Swedish schools decreased by more than half. Table 3 shows some of the studies that analysed the current prevalence of myopia in countries with a so‐called myopia boom; and for comparison, the respective prevalence rates in the Nordic countries Finland, Sweden, Norway and Denmark are listed as well. In all Nordic countries, the current prevalence of myopia in young adults has remained below 30%, while in several Asian countries, it is at least 80% among school leavers. The education systems in the Nordic countries are very similar; they have implemented many of the recommendations outlined in the Strasbourg Manifesto. A comparison of the education systems between countries with high myopia and Nordic countries can help understand why the myopia prevalence of Nordic countries is much lower and what kind of reforms in the school system can reduce the current myopia boom.

**TABLE 3:** Prevalence of myopia in some Asian countries with a high myopia prevalence and in Scandinavian countries.

	Type of study	Refraction with cycloplegia	Age (years)	Prevalence of myopia (%)
**Japan**				
Huang et al. (2019)	First‐year university students	? Self‐reported	19.6 ± 0.9	86.8
Kamei et al. (2025)	A nationwide database study	?	14	83.2
Yotsukura et al. (2019)	Cross‐sectional study of two schools in Tokyo	No	13	84.2
**China**				
Dong et al. (2020)	Systematic review and meta‐analysis	?	16–18	84.8
Wei et al. (2018)	Cross‐sectional university‐based study	Yes	16–26	83.2
**Taiwan**				
Lee et al. (2013)	Army recruits	No	21	86.0
**South Korea**				
Jung et al. (2012)	Army recruits	Yes	19	96.5
**Singapore**				
Woo et al. (2004)	2nd Year medical students	No	19–23	89.8
**Finland**				
Pärssinen et al. (2025)	Army recruits	Yes	19.3 ± 1.55	23.5
**Sweden**				
Bro (2025)	Army recruits	No	17–19	29.0
**Norway**				
Hagen et al. (2018)	Two upper secondary schools	Yes	16–19	13.0
**Denmark**				
Jacobsen et al. (2007)	Conscripts	?	18	12.8

### 4.1 Myopia in some Asian and Scandinavian countries

Table 3 compares the myopia prevalence rates in several Asian and Scandinavian countries, including Finland, Sweden, Norway and Denmark. In all listed studies from Japan, China, Taiwan, South Korea and Singapore, the myopia prevalence exceeded 80%. By contrast, in all four Nordic countries, the prevalence remained below 30%.

The myopia prevalence does not differ substantially among Nordic countries including Finland, and the education systems in Nordic countries are similar in many respects. The first and perhaps most important paragraph of the Strasbourg Manifesto concerned recommendations related to school timetables. When school starts at the age of 7 years, the timetable for a six‐day week during the first two years is recommended that allows for 4 h of lessons per day, of which 3 h should be teaching hours and the remaining 1 h should include singing, exercise or outdoor activities. In Finland, school generally start at the age of 7 years and are preceded by a one‐year, half‐day preschool that prepares children for school. Teaching in preschools is largely play‐based without homework. In Finland, the mean daily time spent at school is 4.0 h in grades 1 and 2, 4.6 h in grades 3 and 4, 5.0 h in grades 5 and 6, and 6.0 h in grades 7–9. A 15‐minute break exists between each 45‐minute lesson for children to go outdoors and play, and for grades 1–6, the school schedule must include at least 2 h per week of outdoor or physical activities (Finnish National Agency of Education, n.d.).

Supplementary private tutoring is practically unknown in Finland; in many Asian countries, most children start school at least one year earlier than in Finland. In those countries, it is common that homework during formal education often starts in preschool years, with 1–2 h of homework per day in the early primary school years. In addition, supplementary private tutoring is common in many Asian countries.

So far, the highest prevalence of myopia has been found among conscripts in South Korea; almost all (96.5%) conscripts were myopic (Kim et al., 2020). The prevalence of myopia in Finnish conscripts in a recent study was 23.5% (Pärssinen et al., 2025). At the age of 7 years, when education usually begins in Finland, the myopia prevalence is only 2%–3% in Finland (Pärssinen, 2012). By contrast, in South Korea (Kim et al., 2020), the myopia prevalence of 7‐year‐old children is already at a level similar or even higher (27%) than that in Finnish conscripts. More worrying are the differences in the prevalence of high myopia in South Korea, in 15‐year‐olds 13%, and in 18‐year‐olds 20%, when in 18‐year‐old Finnish conscript, the prevalence was 0.2% (Pärssinen et al., 2025).

### 4.2 Age of myopia onset

The prevalence of myopia usually begins to increase only after school has started. The earlier the onset myopia is, the faster it progresses, and the stronger myopia is in adulthood (Pärssinen et al., 2021; Pärssinen & Kauppinen, 2019). In a Finnish study, the prevalence of high myopia in adulthood (SE ≤ −6.0 D) was 65% if the onset of myopia was between 8.8 and 9.7 years and only 7% if the onset was between 11.9 and 12.8 years (Pärssinen & Kauppinen, 2019). A Chinese study followed 7‐year‐old children over 2.5 years and found that the progression of myopia was considerably faster among those who had started schooling a year earlier (Liu et al., 2021). Thus, an earlier onset of myopia is associated with a significantly greater risk of high myopia in adults. This is reflected in a high‐myopia prevalence of 20% in 18‐year‐old South Koreans, whereas it is 0.2% in Finns. Early education is likely one of the root causes of the high prevalence of myopia in South Korea.

### 4.3 Fonts and text

Widmark presented one reason for the decrease in myopia prevalence as clearer textbook prints and the replacement of Fraktur with Antiqua font. Table 4 provides an example sentence in different fonts and font sizes.

**TABLE 4:** The same text is written with different fonts and fonts sizes.

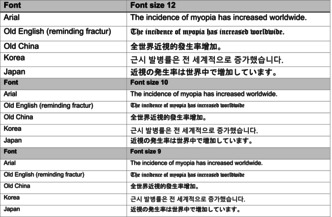

When comparing the same text written in different fonts, the text in Arial seems clearer than that in Fraktur (Old English) used in the 19th century. The differences between these fonts are further emphasized when the text must be written. Naturally, the smaller the text, the closer it should be read to achieve the same visual acuity. The effect of lighting goes in the same direction, as poorer lighting requires a shorter reading distance and a longer reading time. In other words, the clarity of the text and the lighting conditions ought to be such that it would allow reading from a sufficiently long distance, preferably at a distance of approximately 40 cm.

### 4.4 Near work

Numerous studies after the reports by Cohn and Widmark have shown that various forms of near work are an essential cause of myopia (Goldschmidt, 1981; Pärssinen, 1986; Pärssinen & Kauppinen, 2022). A Finnish study compared the relationship between time spent on near work and myopia in 7, 11 and 15‐year‐old school children (Pärssinen & Kauppinen, 2022). If daily near work time was either <1 h or >3 h, the prevalence of myopia was 0.5% vs. 3.9% (seven‐year‐olds), 3.3% vs. 21.2% (11‐year‐olds), and 17.6% vs. 37.2% (15‐year‐olds), respectively (Pärssinen & Kauppinen, 2022). Consequently, outdoor activities reduce the risk of myopia (Morgan et al., 2018; Pärssinen & Kauppinen, 2022; Rose et al., 2008). Nowadays, smart devices are increasingly used. A recent systematic review found that each additional hour of daily digital screen time is associated with a 21% increase in myopia (Ha et al., 2025).

The classification of near work varies depending on research design. Most studies have used questionnaires. Based on the results from a Finnish questionnaire, the subjective assessment of parents that their child reads from a near distance was related to myopia more in 7‐year‐olds (odds ratio, 7.381; 95% confidence interval, 4.054–13.440; *p* < 0.001) than in 15‐year‐olds (odds ratio, 2.237; 95% confidence interval, 1.498–3.057; *p* < 0.001) (Pärssinen et al., 2022). A Chinese questionnaire study showed that a longer reading distance was a preventive factor for myopia (Xiao et al., 2022). In a Finnish 3‐year follow‐up study, the habitual reading distance was measured annually four times (Pärssinen et al., 1989). A shorter average reading distance was related to greater progression of myopia (*r* = −0.220, *p* < 0.001). In the same study, the average reading distance increased from 19 cm at a mean age of 11 years to 27 cm at 14 years of age (Pärssinen & Kauppinen, 2016). A Chinese study found that a reading distance of >33 cm was associated with a lower risk of myopia in female students of primary and middle schools (Jiang et al., 2022). Especially children can easily read at a distance of 10 cm or below. The closer an object, for example a book, is, the narrower the field of peripheral vision outside the book becomes, and the peripheral myopic defocus of that area increases. The distance at which viewing is no longer considered near work is not precisely defined. When the focused object is at a distance of 1 m, the peripheral part of the visual field outside the object is 1 D or less in defocus. For example, watching TV at 4 m distance requires only 0.25 D accommodation. Consequently, watching TV does not increase the risk of myopia (Pärssinen et al., 2014). Thus, it seems reasonable to assume that the association between near work and myopia is stronger when the distance is shorter and the time spent with near work is longer.

### 4.5 Animal experiments

Myopia has been induced in various animal models, for example by using different defocused lenses or occluding or restricting near vision (Wallman & Winawer, 2004). In laboratory experiments with most animal strains, occluding or restricting distant vision leads in an immediate response to refractive changes in the myopic direction and ultimately to deprivation myopia (Schaeffel, 2016). Deprivation myopia develops more easily in younger animals than older animals (Wallman & Adams, 1987; Zhi et al., 2010). The earlier deprivation in monkeys was initiated, and the longer it was maintained, the greater the degree of relative myopia in the deprived eye (Smith 3rd et al., 1987). These observations suggest that parallels exist in the risk factors for myopia, among young children who are reading from a near distance and in young animals with restricted distant vision.

### 4.6 Use of spectacles in near work and posture in reading

**FIGURE 8:**
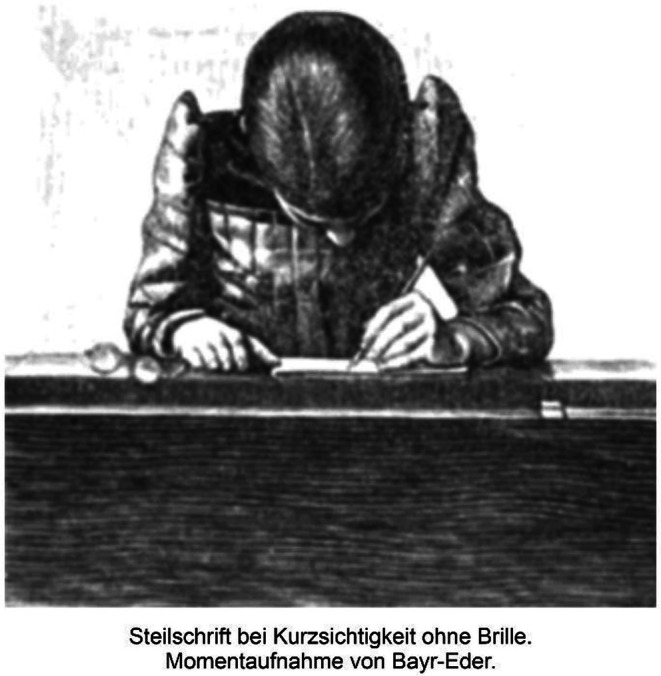
Myopic girl is writing without glasses.

**FIGURE 9:**
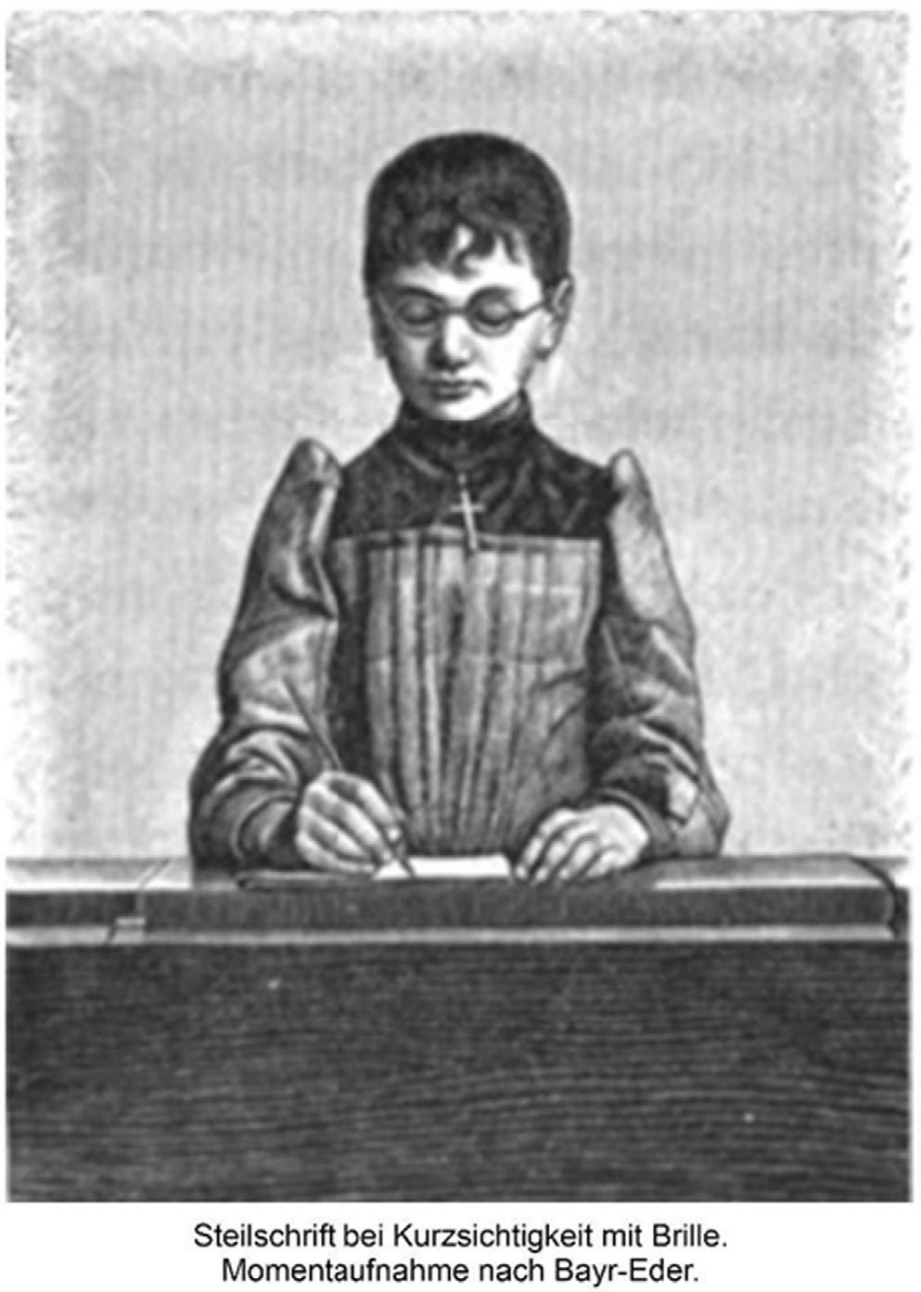
Myopic girl is writing with glasses.

Cohn emphasized the importance of the proportions and size of the school desk so that the posture and distance of near work would be appropriate in relation to the size of the child. He also drew attention to the fact that myopic children often read without spectacles, resulting in poor reading posture and short viewing distances. Figures 8 and 9 illustrate these differences.

Straub (1909) in his article referred to the thesis of Wentink (1908). Wentink studied myopia progression in different age groups regarding different usage of spectacles. Myopic progression was compared between those not wearing spectacles or using under corrected spectacles vs those who used fully corrected spectacles. His study showed that in 7‐ to 13‐year‐olds 14.9% vs. 49%, in 14‐ to 17‐year‐olds 28.5% vs. 49%, and in 18‐ to 21‐year‐olds 57.9% vs. 88.9 % myopia remained stationary regarding the progression of myopia in the non‐corrected or under corrected myopes vs. fully corrected myopes, correspondingly. This indicated that it would be advisable for myopes to wear fully corrected glasses. In the 3‐year treatment trial in 9‐ and 11‐year‐old myopic school children, no difference in the progression of myopia was found between those who wore glasses in near work or did near work without glasses (Pärssinen & Lyyra, 1993). However, a shorter viewing distance, in that study was related to more myopic progression. In the same treatment trial, myopic progression across the 3‐year follow‐up was highest among those who reported that they usually read sitting head downward (−3.58 ± 1.75 D) and lowest among those who usually read on their backs, facing upwards (−2.35 ± 1.53 D, *p* = 0.021). In the same treatment trial, the measured more downwards reading angle was associated with greater myopic progression (*r* = −0.166, *p* = 0.028; Pärssinen & Kauppinen, 2016). Therefore, near work in a sitting position without glasses from short distance may be a potential risk factor for myopia.

### 4.7 Preventive measures

A recent IMI report (Jonas et al., 2021) summarized the current methods that have emerged to reduce or slow the progression of myopia, including the daily application of low‐dose atropine eye drops at concentrations ranging between 0.01% and 0.05%; multifocal spectacle design; contact lenses that have power profiles to induce peripheral myopic defocusing; and orthokeratology contact lenses that are designed to flatten the central cornea, leading to midperipheral steeping and peripheral myopic defocus, during overnight wear to eliminate daytime myopia. The report concluded that compared with other measures, spending more time outdoors is the safest strategy for preventing myopia. Both Cohn and Widmark strongly emphasized the importance of outdoor activities in preventing myopia. Subsequently, several studies have shown that minor time spent outdoors increases the prevalence of myopia and facilitates its progression (Pärssinen, 1986; Pärssinen et al., 2022; Rose et al., 2008). The increase in time spent outdoors was associated with a decrease in myopia in both those who did who did a little or a lot of near (Pärssinen & Kauppinen, 2022). Rose et al. (2008) reported that when children spend more than 2 h per day outdoors, the risk of myopia is reduced, even when they have two myopic parents and continue to perform near work.

In countries with a high prevalence of myopia, efforts have been made to increase the time that schoolchildren spend on outdoor activities. Wu et al. (2013) reported that the incidence of new myopia cases in Taiwan was significantly reduced by approximately 50% after 1 year, when the time spent outdoors was increased by an additional 80 min per day, compared with that of a control group (8.4% versus 17.6%, respectively). In Taiwan, a prospective nationwide cohort study was initiated in 2010 based on the recommendations of 2 h of outdoor activity per day and a 10‐min break after every 30 min of near work. Over the course of the study, the increasing trend of poor uncorrected distant vision (myopia) significantly reversed (Wu et al., 2020). In a study conducted among 6‐year‐old children in China, parents were encouraged to go outdoors after school, on weekends and during holidays, and 40 min of outdoor activities were added to their school days. During a 3‐year follow‐up period, the incidence rate of myopia was significantly lower (30.3%) in the intervention group than in the control group (39.5%) (He et al., 2025).

## 5 LIMITATIONS

There was no standard method for determining myopia, and the definitions have varied between studies. Cohn also used atropine from time to time for cycloplegia but cycloplegia has not generally been used. The lack of cycloplegia, on the other hand, has been compensated for by a stricter definition of myopia. Cohn considered the limit of myopia in his own examinations −1 D in the less myopic eye. Widmark's data is based on measurements by numerous researchers, and there are no detailed reports on how refraction has been determined. When the measurements have been made at the same schools over 20 years, it can be assumed that there would not have been so many differences in the methods that could have significantly distorted the main conclusions about the reduction of myopia.

## 6 CONCLUSION: REDUCTION NEAR WORK DECREASES MYOPIA PREVALENCE

The studies by Cohn, Widmark and numerous others have unquestionably shown the connection between near work and myopia. Without near work, the prevalence of myopia is very low, at a few per cents. The younger the age at which myopia begins, the stronger the myopia in adulthood, and the more frequent myopia‐related eye diseases. Although outdoor activities reduce the incidence and progression of myopia, the most important aspect of myopia prevention is to decrease the amount and intensity of near work, especially during childhood. To implement this aspect would require fundamental changes in the school system, especially in countries where the prevalence of myopia is currently high. Cohn's research, the related Strasbourg Manifesto (1882), and Widmark's observations of the significant decline in myopia at the turn of the 19th and 20th centuries may be helpful tools in considering these issues.

## FUNDING INFORMATION

Eye Foundation Finland, Evald and Hilda Nissi Foundation.

## Supporting information


Supplemantary table 1‐3

Supplement A
