# Supplementary material for: Factors associated with the high prevalence of myopia and its decrease—A historical review
Source: Acta Ophthalmol. 2025 Oct 6;103(8):879–90. doi: 10.1111/aos.70001 (PMC12604449; doi:10.1111/aos.70001)
Supplement: Supplementary file 1 — Supplemantary table 1‐3 [file AOS-103-879-s002.docx]

**Supplemantary table 1-3**

Prevalence of myopia in Germany in the second half of the 1800s.

Copy from Cohn (1882), pages 67-69.

**
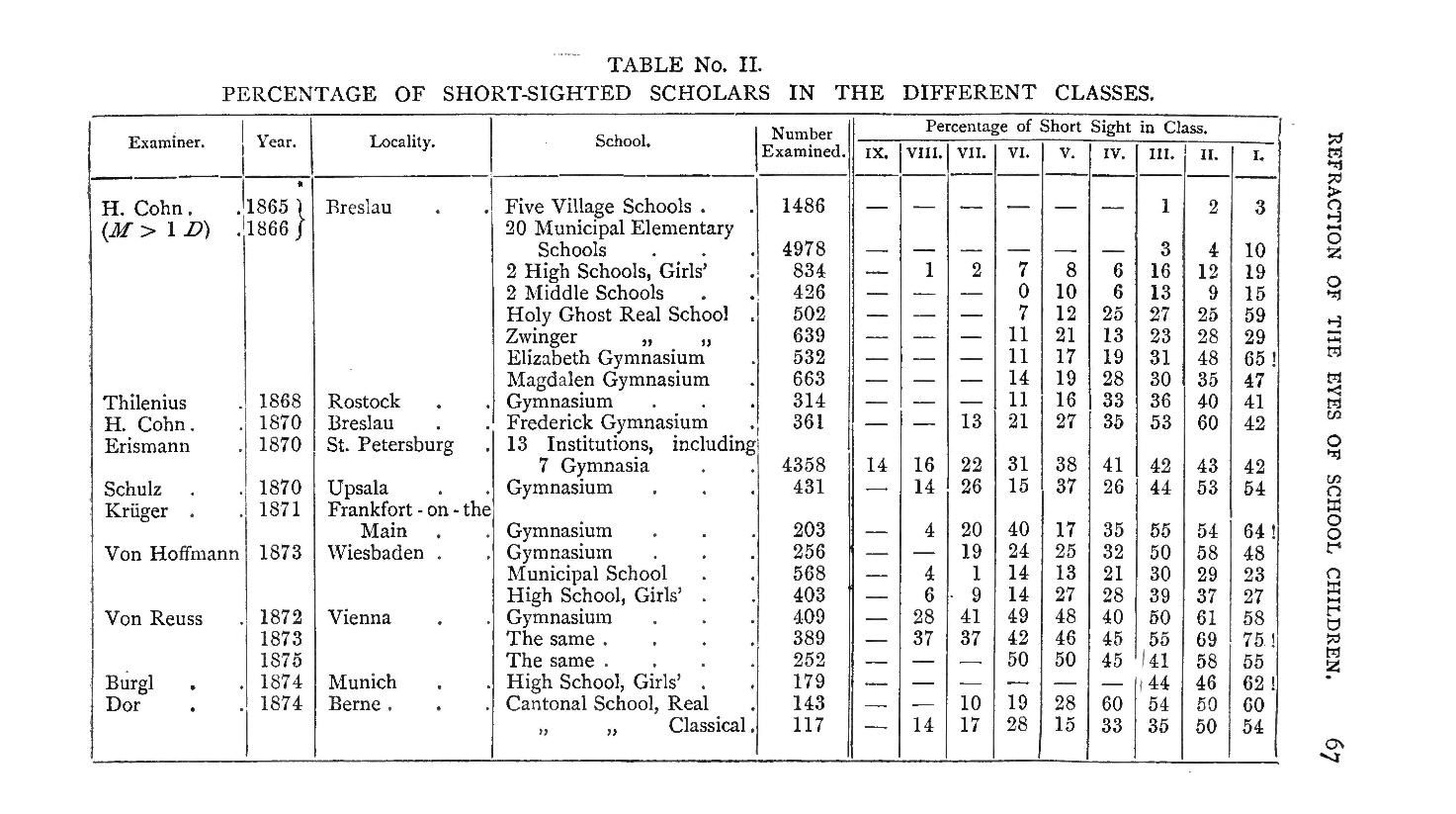
**

**
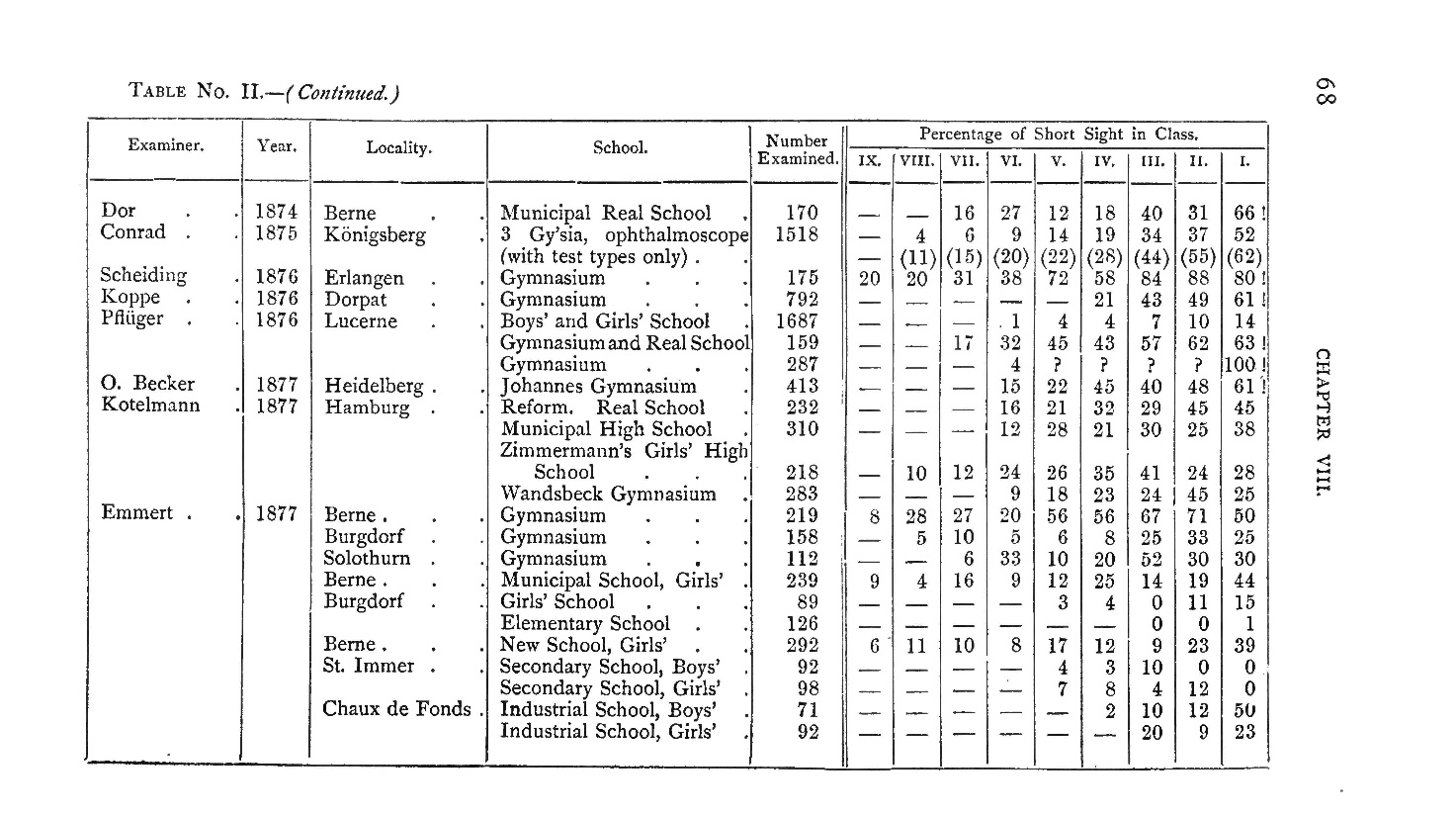
**

**
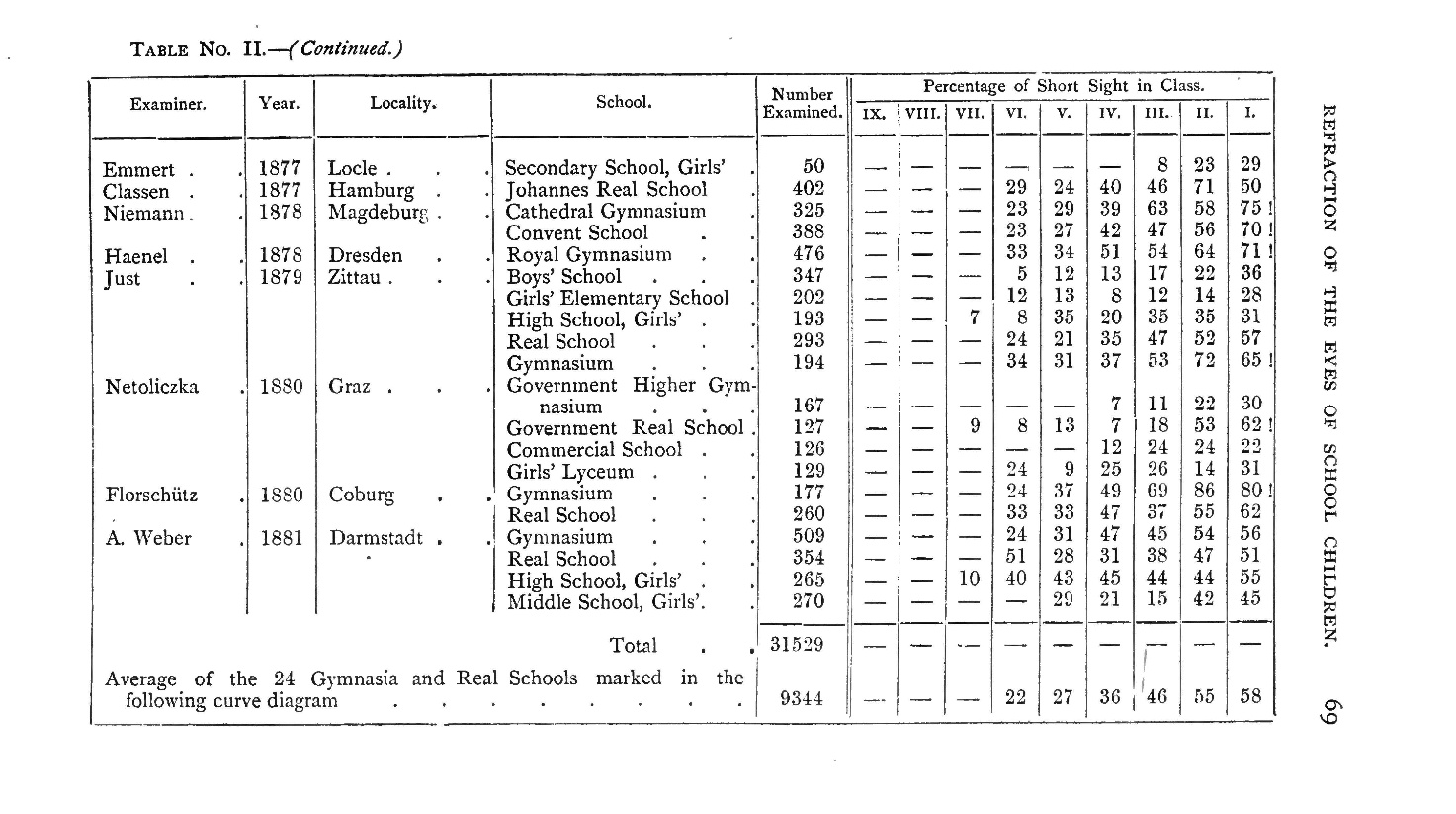
**
