# Supplementary material for: Factors associated with the high prevalence of myopia and its decrease—A historical review
Source: Acta Ophthalmol. 2025 Oct 6;103(8):879–90. doi: 10.1111/aos.70001 (PMC12604449; doi:10.1111/aos.70001)
Supplement: Supplementary file 2 — Supplement A [file AOS-103-879-s001.docx]

**Supplement A**

(Quoted, translated and edited from Cohn (1892), page 504-507

**Recommendations of the Strasbourg Commission for the organisation of school education**

TABLE 1 Paragraph 1 of the Strasbourg Manifesto about the arrangement of school education, focusing on its weekly content. Quoted and edited from Cohn (1882), p. 504

| Age years | Grades of school | Teaching hours | Singing hours | Gymnastic hours | Homework hours | Total |
| --- | --- | --- | --- | --- | --- | --- |
| 7 and 8 | 1 and 2 | 18 | 1 | 2 - 2.5 | 3 | 24.0–24.5 |
| 9 | 3 | 20 | 1 | 2 - 2.5 | 5-6 | 28-29.5 |
| 10 and 11 | 4 and 5 | 24 | 2 | 2 -3 | 8 | 36 -37 |
| 12, 13, 14 | 6 and 7 | 26 | 2 | 2 | 12 | 42 |
| 15 to 18 | 8 and 9 | 30 | 2 | 2 | 12–18 | 46.0–52.0 |

2. Incorporate a 10-minute pause after every 2 hours of lesson time—both in the morning and afternoon. If more than two lesson hours are consecutive, incorporate a pause of 15 minutes between the 2^nd^ and 3^rd^ lessons and 20 minutes between the 4^th^ and 5^th^ lessons.

3. Adopt one half-day holiday (afternoon) during the week and another at the end of the week.

4. Assign no tasks between the morning and afternoon of the same day, with Sunday left entirely free from schoolwork.

5. Extend autumn holidays to begin in early August and continue until mid-September. Assign no tasks during the Whitsuntide and Christmas holidays.

6. Arrangements regarding summer holidays are judicious and should continue to do so.

7. The maximum permissible number of scholars in each class should be in line with Pettenkofer's rule. According to Pettenkofer, in every lesson hour, each scholar would require a supply of 60 cubic meters of air.

8. Use emulation within limits, abandon the one-sided principle of prioritising extempore performances, and avoid the over-exertion of scholars while preparing for the final examination.

9. Schedule lessons demanding reflection and memory in the morning.

10. In addition to the obligatory gymnastic lessons, introduce swimming, outdoor games, excursions and skating to scholars. Dedicate a total of 8 hours per week to bodily exercises.

11. For effective lighting in classrooms of new higher school buildings with less than 5 meters] wide, add a single row of windows to the left of the scholars. For rooms wider than the above measurements, enable lighting from both sides. In exceptional cases, consider lighting from behind the scholars as well.

12. For rooms with one-sided lighting, ensure that the rooms receive light from the east, west, or north.

13. For existing school buildings, avoid teaching in rooms that receive light only from the south.

14. For rooms that lack sufficient lighting, focus on bevelling windows, creating niches and adding new windows in wall spaces.

15. Avoid using rooms with insufficient natural light, especially corner rooms in square courtyards, as classrooms.

16. Introduce roller blinds and sufficient appliances for artificial lighting in all classrooms.

17. The school desks should be so well-placed that direct sun light reaches every scholar. Avoid using the part of the room thrown into shadow by wide wall spaces between windows.

18. There should be no highly reflecting surfaces, such as white walls, near school buildings.

19. Replace all faultily constructed desks, without exception and at the earliest, with ergonomically and rationally constructed ones.

20. Tests the size of letters, form of typography and distance between letter and letter, word and word and line and line in schoolbooks, charts and atlases. Books that fail to conform to the above requirements (entirely in accordance with those I have proposed above in Chapter XVIII) should be gradually removed from the school.

21. Timetables should be designed systematically to effectively manage the occupations of the scholars and, in particular, avoid the protraction of reading through several consecutive hours.

22. Short-sighted scholars should be seated in the front rows and well-lit areas and relieved from all tasks that could strain their eyes. Stamping and fine-tuning of maps or geometric patterns should be avoided.

23. Regulations guiding building plans, furniture and equipment should be issued for higher schools as well.

24. Designs for building alteration or new construction of a higher school should be examined and reported on with reference to these regulations by a medical expert officially appointed for the purpose.
